# Identification and Transcriptome Analysis of a Novel Allelic Mutant of *NAL1* in Rice

**DOI:** 10.3390/genes15030325

**Published:** 2024-03-02

**Authors:** Yang Wang, Wanxin Xu, Yan Liu, Jie Yang, Xin Guo, Jiaruo Zhang, Jisong Pu, Nenggang Chen, Wenfeng Zhang

**Affiliations:** 1 College of Agricultural Science, Panxi Crops Research and Utilization Key Laboratory of Sichuan Province, Xichang University, Liangshan 615013, China; xin1473692003@163.com (W.X.); liuy6120420@163.com (Y.L.); y02528c@163.com (J.Y.); gx01148022@163.com (X.G.); zjruo12304@163.com (J.Z.); xcp106789331@163.com (J.P.); 18683930792@163.com (W.Z.); 2 State Key Laboratory of Crop Gene Exploration and Utilization in Southwest China, Sichuan Agricultural University, Chengdu 611130, China; 3 Institute of Crop Germplasm Resources, Guizhou Academy of Agricultural Sciences, Guiyang 550006, China; chennenggang123@163.com

**Keywords:** rice, narrow leaf allelic mutant, gene cloning, *NAL1*, RNA-Seq

## Abstract

Leaf morphology is a crucial aspect of plant architecture, yet the molecular mechanisms underlying leaf development remain incompletely understood. In this study, a narrow leaf mutant, *m625,* was identified in rice (*Oryza sativa* L.), exhibiting pleiotropic developmental defects. Pigment measurement revealed reduced levels of photochromic pigments in *m625*. Cytological analysis demonstrated that the *m625* gene affected vascular patterns and cell division. Specifically, the narrowing of the leaf was attributed to a decrease in small vein number, shorter vein spacing, and an abnormal V-shaped arrangement of bulliform cells, while the thickening was caused by longer leaf veins, thicker mesophyll cells, and an increased number of parenchyma cell layers. The dwarf stature and thickened internode were primarily due to shortened internodes and an increase in cell layers, respectively. Positional cloning and complementation assays indicated that the *m625* gene is a novel allele of *NAL1*. In the *m625* mutant, a nucleotide deletion at position 1103 in the coding sequence of *NAL1* led to premature termination of protein translation. Further RNA-Seq and qRT-PCR analyses revealed that the *m625* gene significantly impacted regulatory pathways related to IAA and ABA signal transduction, photosynthesis, and lignin biosynthesis. Moreover, the *m625* mutant displayed thinner sclerenchyma and cell walls in both the leaf and stem, particularly showing reduced lignified cell walls in the midrib of the leaf. In conclusion, our study suggests that *NAL1*, in addition to its known roles in IAA transport and leaf photosynthesis, may also participate in ABA signal transduction, as well as regulate secondary cell wall formation and sclerenchyma thickness through lignification.

## 1. Introduction

Leaf architecture, as one of the important agronomic traits, regulates photosynthesis, respiration, and transpiration, thereby impacting plant growth and development [1,2,3]. In rice, the top three leaves are considered to be the mainly source of carbohydrates that accumulate in the grains and are proposed to be long, erect, narrow, rolled, and thick in super-high-yielding hybrid rice [4,5]. Moderate leaf narrowing can increase dry matter accumulation by enhancing light capture, carbon fixation, and gas exchange during photosynthesis. Hence, identifying narrow leaf mutants and isolating genes associated with narrow leaf characteristic would be advantageous for genetic improvement and ideotype breeding in rice.

To date, numerous rice mutants related to narrow leaf have been discovered, with a reduction in leaf vein number, especially small veins being an obvious peculiarity [6,7,8,9]. It has been found that some narrow-leaf genes are related to plant hormones, particularly the IAA and GA pathways. For instance, *NAL1,* a putative trypsin-like serine and cysteine protease gene, regulates leaf vascular patterns, polar auxin transport, and leaf lateral outgrowth. Mutants with defects in *NAL1* exhibit narrow leaves due to a significant reduction in the capacity of polar auxin transport [10,11]. *NAL7* encodes a flavin monooxygenase and participates in tryptophan-dependent IAA biosynthesis. Its mutation leads to decreased IAA levels in the narrow leaf mutant *nal7* [12]. Paralogs *NAL2* and *NAL3* encode the same transcriptional activator OsWOX3A protein, which influences the development of multiple organs, including the formation of vascular bundles and leaf lateral-axis outgrowth [13]. Moreover, *OsWOX3A* is a GA-responsive gene involved in the feedback regulation of GA biosynthesis to maintain GA homeostasis in rice. The double mutant *nal2/3* displays extremely narrow leaves, while overexpressing transgenic plants exhibit wide leaves [14]. Additionally, some narrow-leaf genes are also connected to cell wall formation, such as *NRL1*, which encodes the cellulose synthase-like D4 protein and is responsible for cell wall biosynthesis and plant growth. In the *nd1* mutant, the primary cell walls in stem and root tip cells exhibit structural defects [15,16]. *NRL2* encodes a novel plant-specific protein and mediates leaf development by influencing secondary cell wall formation and phenylalanine metabolism. Its deficiency results in pleiotropic effects, including reduced leaf width and vein number, as well as impaired differentiation of abaxial sclerenchyma cells [3,17]. In addition, other genes, such as *NAL9* regulating chloroplast biosynthesis, are also responsible for the regulation of narrow leaf [18]. Despite the identification of an increasing number of narrow-leaf genes in rice, the underlying regulatory mechanisms of leaf development remain unclear. 

To improve comprehension of the genetic underpinnings governing leaf morphology, here we reported a rice narrow leaf mutant *m625*. It displayed a narrow-leaf phenotype due to a decrease in small vein number, shorter vein spacing, and an abnormal V-shaped arrangement of bulliform cells. Gene cloning and complementation assays revealed that the *m625* gene is a novel allelic variant of *NAL1.* Through pigment measurement, paraffin section, RNA-Seq, and qRT-PCR analyses, we uncover new findings concerning the regulatory roles of *NAL1* in rice growth and development. The present results suggest that apart from its known functions in IAA transport and leaf photosynthesis, *NAL1* may also be involved in ABA signal transduction, as well as regulating secondary cell wall formation and sclerenchyma thickness through its impact on lignification. 

## 2. Materials and Methods

### 2.1. Plant Materials and Growth Conditions

The mutagenesis library was constructed using ^60^Co radiation on Qiaogangzhenzhu, a local *japonica* rice variety in Guizhou province. A narrow leaf mutant, named *m625*, was screened from this library. For genetic analysis, the F_2_ population was used through crossing *m625* with its wild parent Qiaogangzhenzhu. For gene mapping, the F_2_ population was obtained through crossing *m625* with an indica restorer line R100. Plants were cultivated in the local fields in Xichang (latitude 27°57′ N, longitude 102°12′ E, and altitude 1531.1 m), Liangshan Yi Autonomous Prefecture, Sichuan, China. 

### 2.2. Pigment Measurement

The photosynthetic pigment contents in the latest fully extended leaves were measured using an acetone extraction method at both the seedling and heading stages. Each sample containing 0.2 g of fresh leaves was immersed in an 80% acetone solution for 48 h. Afterwards, the samples were adjusted to a volume of 25 mL and measured using a UV-6000 UV–visible spectrophotometer (Metash, Shanghai, China) at three specific wavelengths (470 nm, 663 nm, and 646 nm). The entire process was conducted in darkness. The contents of chlorophylls and carotenoids were analyzed based on a calculation method described by Lichtenthaler and Wellburn [19]. 

### 2.3. Cytological Analysis

Paraffin sections were prepared as in the procedure described by Li et al. [20] to examine the morphological characteristics of tissues and cells. Leaves at the booting and heading stages, as well as stems at the heading stage, were individually fixed using a 50% formaldehyde–acetic acid–ethanol fixative (FAA) solution (Servicebio, Wuhan, China). Given the firmness of the stems, a softening process was necessary to facilitate slicing [21]. As such, we softened the stems with an ethylenediamine solution before slicing. After dehydration, transparency, embedding, sectioning, and staining, cross-sections of all samples were observed using a DS-U3 light microscope (Nikon, Tokyo, Japan). All samples were stained in accordance with the saffron-O and Fast Green Stain Kit (For Plant) (Solarbio, Beijing, China).

### 2.4. Gene Mapping and Marker Development

Two hundred and sixty recessive plants displaying the narrow-leaf phenotype from the (*m625* × R100) F_2_ segregating population were chosen for gene mapping. Individual extractions of the whole genome DNA were carried out for *m625*, R100, and the 260 mutant individuals to facilitate linkage mapping analysis. Initially, over 300 pairs of simple sequence repeat (SSR) markers were screened for initial mapping. Subsequently, insertion/deletion (InDel) markers were designed for fine mapping according to genome sequence polymorphism between *japonica* and *indica* (Table S1). To expedite the identification of the *m625* gene, leaves from 30 WT plants and 30 mutant plants from the (*m625* × Qiaogangzhenzhu) F_2_ population were sent to Novogene Biotech Co., Ltd. (Beijing, China) for whole-genome sequencing. The MutMap analysis was performed between *m625* and WT using Nipponbare as the reference genome sequence [22]. 

### 2.5. Complementation Analysis

To complement the *m625* mutant, a 1749 bp cDNA sequence of *LOC_Os04g52479* was acquired from Qiaogangzhenzhu using high-fidelity polymerase and the primers *M625*-CoF: 5′-GGTACCCGGGGATCCCTGCAGATGAAGCCTTCGGACGATAA-3′ and *M625*-CoR: 5′-TGCCTGCAGGTCGACCTGCAGTCATTTCTCCAGGTCAAGGC-3′ (both *M625*-CoF and *M625*-CoR contained the *XbaI* site). The resultant cDNA fragment was constructed to the vector pCAMBIA2300 carrying the rice *Actin 1* promoter through homologous recombination. The plasmid pC2300-*M625* was delivered into *m625* via *Agrobacterium*-mediated genetic transformation. The complementary transgenic lines were identified using specific primers *M625*-TraF/R, which targeted the *M625* gene and the pCAMBIA2300 vector, respectively (Table S1). 

### 2.6. RNA-Seq Analysis

Leaves at the tillering and the top three leaves at the heading stage, as well as stems at the heading stage, were used to extract total RNA using an RNA extraction kit (Qiagen, Hilden, Germany). Construction and sequencing of cDNA libraries were performed by Novogene Biotech Co., Ltd. (Beijing, China). Identification of the differentially expressed genes (DEGs) were carried out following a previously described method [23,24]. DEGs were subjected to the Kyoto Encyclopedia of Genes and Genomes (KEGG) analysis using KEGG databases [25]. 

### 2.7. qRT-PCR Analysis

Leaves at the tillering and the top three leaves at the heading stage, as well as stems at the heading stage, were used to extract total RNA using an RNA isolator kit (Vazyme, Nanjing, China). Reverse transcription was performed on the extracted RNA using a reverse transcription kit from the same manufacturer. The cDNA was utilized for qRT-PCR analysis employing a real-time PCR system (Bio-Rad, Berkeley, CA, USA), with *Actin 1* serving as an internal control. The expression levels of five genes related to IAA and ABA signal transduction, four genes associated with photosynthesis, and seven genes involved in phenylpropanoid biosynthesis were analyzed through qRT-PCR. The qRT-PCR primers can be found in Table S2.

## 3. Results

### 3.1. Phenotypic Characterization of the m625 Mutant 

**Figure 1:**
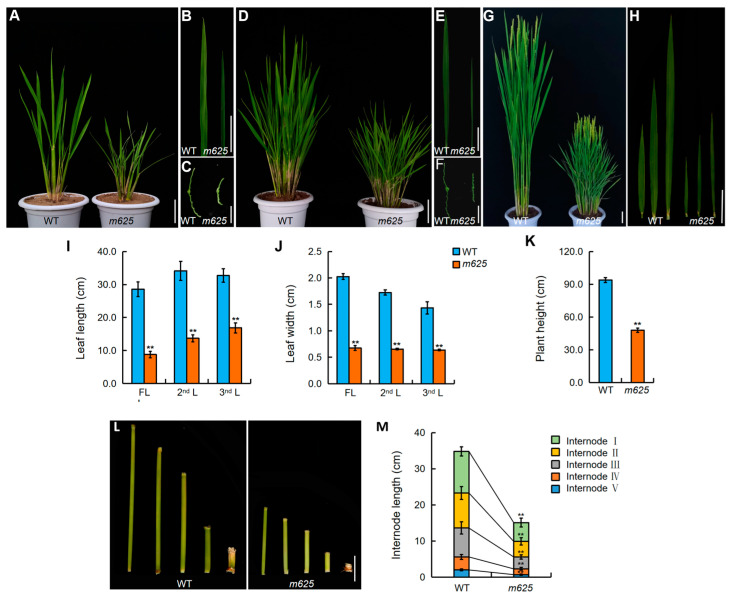
Phenotypic characterization of wild-type (WT) and *m625*. (**A**,**D**,**G**) Plant architecture at the tillering, elongation and heading stages, respectively. Scale bars, 5 cm. (**B**,**E**) Leaf morphology at the tillering and elongation stages, respectively. Scale bars, 5 cm. (**C**,**F**) Transverse section of the leaves at the tillering and elongation stages, respectively. Scale bars, 5 mm. (**H**) Morphology of the top three leaves at the heading stage. Scale bar, 5 cm. (**I**,**J**) Statistical analysis of the length and width of the top three leaves. Data are means ± SD (*n* = 24). The blue and orange columns represent WT and *m625* in the histogram, respectively, the same as below. (**K**) Statistical analysis of plant height. Data are means ± SD (*n* = 24). (**L**) Characteristics of internodes (I–V) in WT and *m625*. Scale bar, 2 cm. (**M**) Quantification of WT and *m625* internode length. Data are means ± SD (*n* = 9). Asterisks represent significant differences using Student’s *t*-test (** *p* < 0.01) in (**I**–**K**,**M**).

The *m625* mutant, derived from the Guizhou landrace rice Qiaogangzhenzhu (*japonica* cv.) through radiation mutagenesis, exhibited narrow leaves and short plant height during the tillering stage, which became more pronounced after heading (Figure 1A–H). At the heading stage, the lengths of the top three leaves in *m625* were significantly reduced by 69.2%, 59.9%, and 48.5%, with a corresponding dramatic reduction in leaf width by 66.7%, 62.1%, and 55.5%, respectively (Figure 1I,J). By maturity, *m625* was around 50% shorter in plant height compared to the WT (Figure 1K). To explore the cause of dwarfism, we further measured the length of internodes I–V. The results showed that the dwarfing phenotype in *m625* was due to the shortening of each internode (Figure 1L,M). Additionally, other agronomic traits were also examined. Apart from the seed setting rate, *m625* exhibited a significant increase in effective panicle number but decreases in panicle length, primary branch number, secondary branch number, and grain number per panicle (Figure S1). These observations strongly suggest that *m625* displays pleiotropic developmental defects.

### 3.2. Pigment Contents of the m625 Mutant

**Figure 2:**
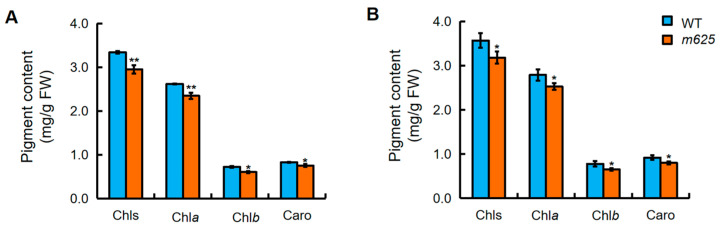
Pigment contents in leaves of WT and *m625*. (**A**) The levels of pigments in leaves at the seedling stage. (**B**) The levels of pigments in leaves at the booting stage. Chls, total chlorophyll; Chl*a*, chlorophyll *a*; Chl*b*, chlorophyll *b*; Caro, carotenoids. Data are means ± SD (*n* = 9). Asterisks represent significant differences using Student’s *t*-test (* *p* < 0.05; ** *p* < 0.01).

For analyzing the impact of the narrow leaf mutation in *m625* on photosynthesis, the levels of photosynthetic pigments were examined at different growth stages. The *m625* mutant showed a notable reduction in total chlorophyll, chlorophyll a, chlorophyll b, and carotenoids compared to the WT at the seedling and heading stages (Figure 2). This indicates that the narrow-leaf characteristic of *m625* influences the photosynthetic pigment levels in leaves.

### 3.3. Cytological Characteristics of the m625 Mutant

**Figure 3:**
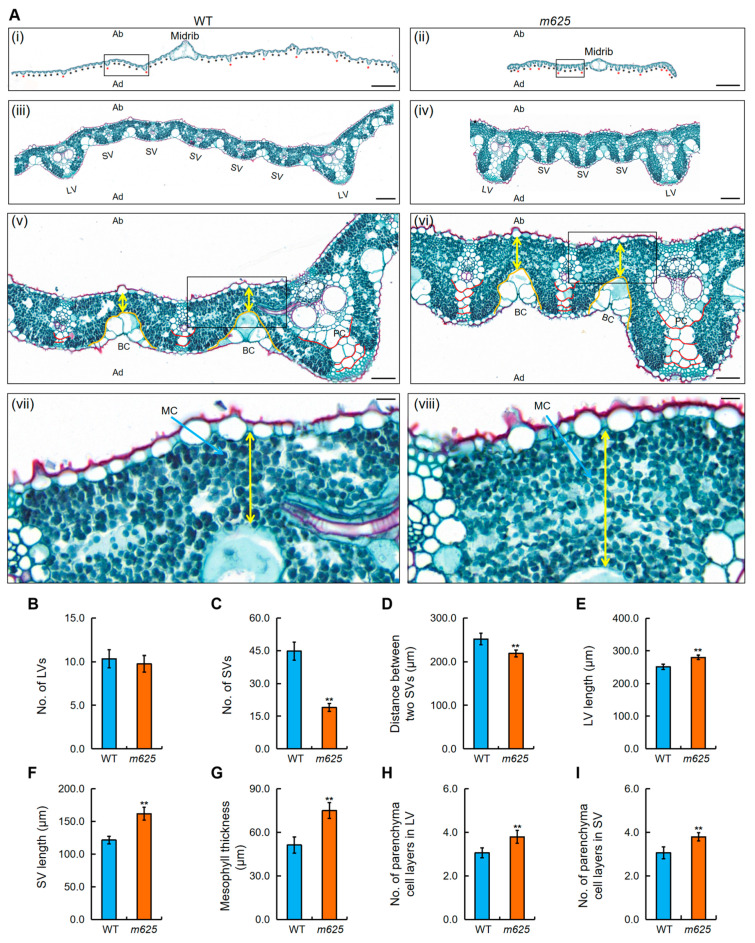
Cytological analysis of the leaf in WT and *m625*. (**A**) Transverse section of the leaf at the booting stage. (**i**,**ii**) Appearance comparison of the leaf. Red and black asterisks denote large vein (LV) and small vein (SV), respectively. (**iii**,**iv**) Comparison of LV number, SV number, and vein spacing. (**iii**) and (**iv**) are the zoomed-in images of black rectangles in (**i**) and (**ii**), respectively. (**v**,**vi**) Comparison of bulliform cells, mesophyll thickness, and number of parenchyma cell layers in veins. Orange curves outline the shape of bulliform cells. Yellow double-headed arrows represent mesophyll thickness. Red curves outline the parenchyma cell layers. (**vii**,**viii**) Enlarged images of mesophyll thickness. Blue arrows denote mesophyll cells. Ab, abaxial surface. Ad, adaxial surface; BC, bulliform cell; MC, mesophyll cell; PC, parenchyma cell. Scale bars, 1000 μm (**i**,**ii**); 100 μm (**iii**,**vi**); 50 μm (**v**,**vi**); 10 μm (**vii**,**viii**). (**B**–**I**) Numerical comparison of LV number, SV number, distance between two SVs, LV length, SV length, mesophyll thickness, and number of parenchyma cell layers in LVs and SVs. Data are means ± SD from five leaves. Asterisks represent significant differences using Student’s *t*-test (** *p* < 0.01).

To investigate the cause of leaf narrowing, paraffin sections of the leaves were produced. When comparing the leaf vascular systems of *m625* and the WT, we found no significant difference in large vein (LV) number. However, there was a significant decrease in small vein (SV) number in *m625* (Figure 3A–C). The WT typically had five to six SVs between LVs, whereas *m625* only had two to three SVs, with a noticeably shorter distance between them (Figure 3A,D). Moreover, the bulliform cells (BCs) in the WT exhibited a slight U-shaped arrangement, while those in *m625* showed a slight V-shape (Figure 3A). These findings suggested that the narrow leaf in *m625* was attributed to a decreased number of SVs, shorter vein spacing, and an abnormal V-shaped arrangement of BCs. Another notable characteristic of *m625* was its thickened leaves. Based on a series of cross-sectional images, we observed remarkable increases in the length of LVs and SVs in *m625* leaves (Figure 3A,E,F). Subsequently, we quantified the mesophyll thickness and the number of parenchyma cell layers. The mesophyll thickness in *m625* was significantly higher, indicating an increase in the number of mesophyll cell layers in *m625* (Figure 3A,G). Additionally, the *m625* leaves exhibited four layers of parenchyma cells within LVs and SVs, while most wild-type leaves contained two to three layers (Figure 3A,H,I). Therefore, the above results demonstrate that the *m625* gene affects the vascular pattern and cell division in the leaf. 

**Figure 4:**
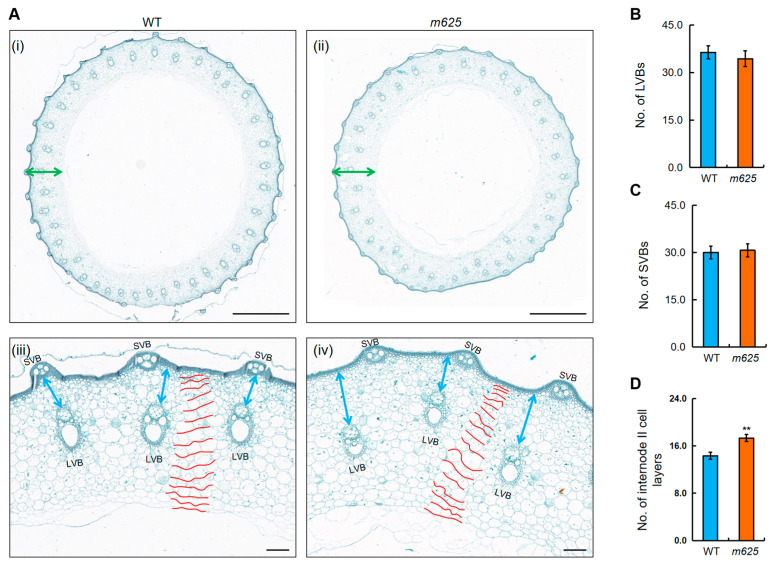
Cytological analysis of the stem in WT and *m625*. (**A**) Transverse section of internode II at the heading stage. (**i**,**ii**) Appearance comparison of internode II. Green double-headed arrows denote the internode thickness. (**iii**,**iv**) Zoomed-in images of transverse section. Blue double-headed arrows show the distance from the large vascular bundle to the outer ring. Red curves outline the internode II cell layers. LVB, large vascular bundle; SVB, small vascular bundle. Scale bars, 1000 μm (**i**,**ii**); 100 μm (**iii**,**iv**). (**B**–**D**) Numerical comparison of LVB number, SVB number, and cell layer number in internode II. Data are means ± SD from three biological replicates. Asterisks represent significant differences using Student’s *t*-test (** *p* < 0.01).

To investigate whether this mutation has a similar effect on the stem as on the leaf, paraffin sections of internode II of the main stem were produced. Although the number of large and small vascular bundles in *m625* did not show significant changes, we observed alterations in the distribution and size of large vascular bundles (LVBs) (Figure 4A–C). In the cross-sections of the WT internode II, LVBs were well-organized, whereas in *m625*, LVBs were smaller and disorganized, and were located closer to the inner ring and further from the epidermis (Figure 4A). Subsequently, we manually recorded the cell layer number and found that the average number of cell layers in internode II of *m625* was significantly higher (Figure 4D). These findings suggest that the thickened internode in *m625* resulted from an increase in cell layers. Therefore, the *m625* gene has a similar effect on the vascular pattern and cell division in the stem.

### 3.4. Cloning of the m625 Gene 

For genetic analysis, the F_2_ (*m625* × Qiaogangzhenzhu) population was constructed. All F_1_ plants displayed a normal phenotype, while the narrow-leaf phenotype in the F_2_ population followed a segregation ratio of 3:1 (Table S3), indicating that a single Mendelian factor controlled the narrow-leaf phenotype of the mutant *m625*. 

**Figure 5:**
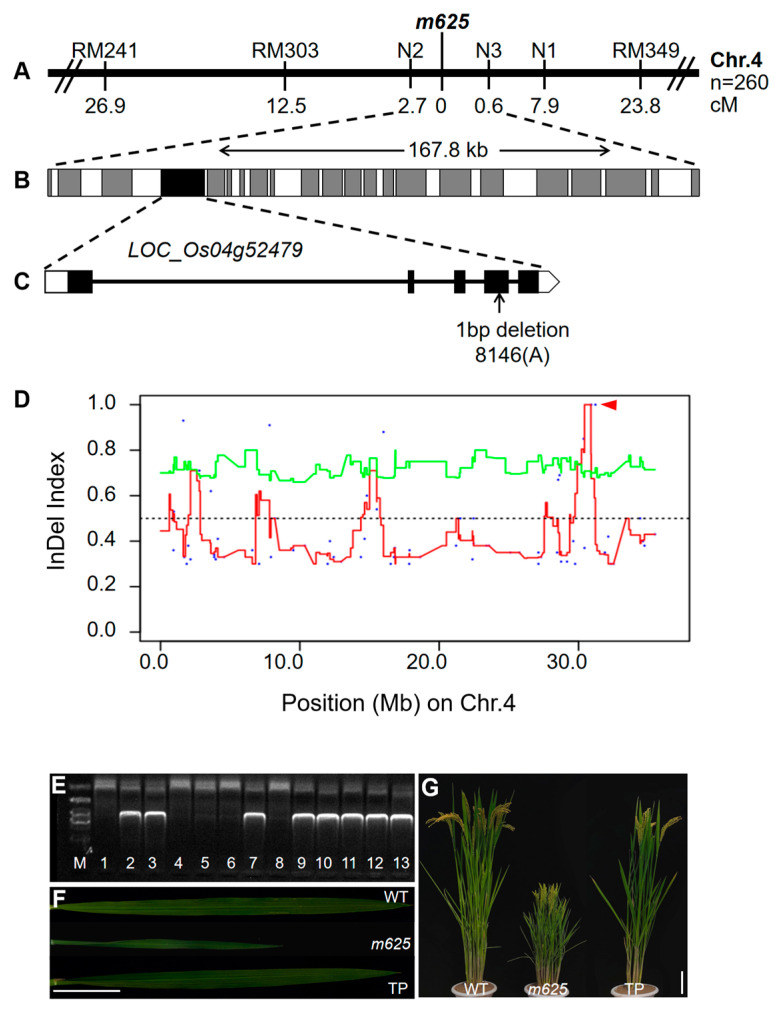
Gene cloning and function verification of *m625*. (**A**) The *m625* gene was located in a 167.8 kb region between N2 and N3 on chromosome 4 using 260 homozygous F_2_ plants. (**B**) The 167.8 kb region contains 22 genes, and the candidate gene *LOC_Os04g52479* was indicated with the black box. (**C**) The structure of *LOC_Os04g52479* contains five exons and four introns. The black arrow represents a single nucleotide deletion at position 8146 bp in *LOC_Os04g52479* in *m625*. (**D**) InDel index plots for *m625* showed genetic linkage on chromosomes 4, in which a nucleotide deletion (InDel index value of 1.0) was found in 167.8 kb region. This deletion was positioned in the fourth exon region of *LOC_Os04g52479*, as indicated with the red triangles. (**E**) PCR identification of positive transgenic plants. M, DL-2000 marker; 1, *m625* (negative control) 2, pC2300-*M625* plasmid (positive control); 3–13, transgenic plants. (**F**) Leaf morphology in WT, *m625*, and TP (transgenic plants) at the booting stage. Scale bar, 5 cm. (**G**) Plant architecture of WT, *m625*, and TP during the grain filling stage. Scale bar, 10 cm.

To fine map the *m625* locus, a total of 260 individuals with the narrow leaf trait were selected from the F_2_ (*m625* × R100) population. The initial mapping indicated that the *m625* gene was positioned between the SSR markers RM241 and RM349 on the long arm of chromosome 4. Then, one SSR and three InDel markers (Table S1) were developed to further narrow down the *m625* gene to a 167.8 kb region between N2 and N3 (Figure 5A). Within this region, 22 putative genes have been annotated by the Michigan State University (MSU) Rice Genome Annotation Project, including a narrow-leaf gene *NAL1* (*LOC_Os04g52479*), which has been cloned (Figure 5B; Table S4). Additionally, we performed whole-genome resequencing on 30 normal plants from the WT parent and 30 narrow-leaf plants from the F_2_ (*m625* × Qiaogangzhenzhu) population. MutMap analysis identified a nucleotide deletion with an InDel index value of 1.0 in this region. This deletion was found in the fourth exon region of *LOC_Os04g52479* (Figure 5D). Therefore, *LOC_Os04g52479* was firstly considered as the candidate gene for *m625*. 

Subsequently, the *LOC_Os04g52479* gene was amplified and sequenced in *m625* and the WT, confirming a single nucleotide deletion at position 8146 (A) in the genomic sequence of *LOC_Os04g52479* in *m625*, consistent with the MutMap analysis. Moreover, cDNA sequencing analysis revealed a nucleotide deletion at position 1103 in the cDNA of this gene (Figure 5C), causing a change in the amino acid at position 368 from Asp to Ala, and affecting the translation of all subsequent amino acids. Ultimately, a stop codon was formed at amino acid position 391, resulting in premature termination of protein translation. 

### 3.5. Complementation of the m625 Mutant

For confirming that the narrow-leaf phenotype of *m625* was due to the mutation in the *NAL1* (*LOC_Os04g52479*) gene, functional complementation was conducted by introducing the cDNA of *NAL1* from Qiaogangzhenzhu driven by the rice *Actin 1* promoter into *m625*. Seven independent transgenic lines possessed positive PCR bands (Figure 5E) and displayed a normal phenotype similar to the WT (Figure 5F,G and Figure S2), demonstrating that *NAL1* is the candidate gene responsible for the mutation in *m625*. 

### 3.6. Transcriptome Analysis of the m625 Mutant

To further understand the biological function of *NAL1*, RNA-Seq experiments were conducted. A total of 571 DEGs were identified in the leaf at the tillering stage, with 164 genes upregulated and 407 genes downregulated. Moving on to the heading stage, 676 DEGs were found in the leaf, with 310 genes upregulated and 366 genes downregulated. Similarly, 746 DEGs were identified in the stem, with 469 genes upregulated and 277 genes downregulated (Figure S3; Table S5). Subsequently, based on the function annotations of DEGs, we completed KEGG analysis in different groups. 

**Figure 6:**
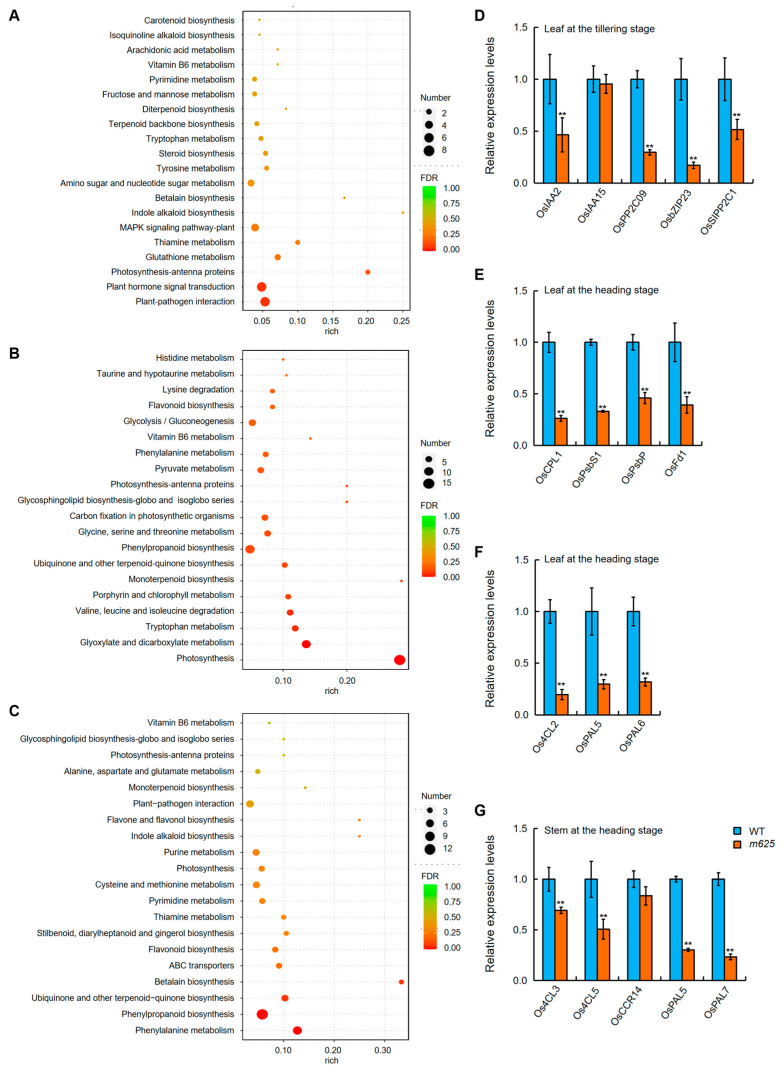
RNA-Seq and qRT-PCR analyses among WT and *m625*. (**A**) KEGG analysis of DEGs in the leaf at the tillering stage. (**B**) KEGG analysis of DEGs in the leaf at the heading stage. (**C**) KEGG analysis of DEGs in the stem at the heading stage. (**D**) Expression analysis of DEGs associated with IAA and ABA signal transduction in the leaf at the tillering stage. *OsIAA2* and *OsIAA15* are auxin-responsive Aux/IAA family genes; *OsPP2C09*, *OsbZIP23*, and *OsSIPP2C1* are ABA signal regulator genes. (**E**) Expression analysis of DEGs associated with photosynthesis in the leaf at the heading stage. *OsCPL1* is a plastocyanin gene; *OsPsbS1* and *OsPsbP* are photosystem II genes; OsFd1 is a photosynthetic ferredoxin gene. (**F**,**G**) Expression analysis of DEGs associated with phenylpropanoid biosynthesis in the leaf and stem at the heading stage, respectively. *Os4CL2*, *Os4CL3* and *Os4CL5* are 4-Coumarate:Coenzyme A Ligase genes; *OsCCR14* is a cinnamoyl-CoA reductase gene; *OsPAL5*, *OsPAL6*, and *OsPAL7* are phenylalanine ammonia–lyase genes. Data in (**D**–**G**) are means ± SD from three biological replications. Asterisks represent significant differences using Student’s *t*-test (** *p* < 0.01).

In the leaf at the tillering stage, we found that the pathways related to plant hormone signal transduction and plant–pathogen interaction had the highest number of DEGs (Figure 6A; Table S6). Given previous studies highlighting the function of *NAL1* in regulating polar auxin transport [10,11], we specifically focused on the plant hormone signal transduction pathway. Within this pathway, the DEGs were mainly involved in IAA and ABA signaling, such as *OsIAA2* and *OsIAA15* (auxin-responsive Aux/IAA gene family members) [26], as well as *OsPP2C09*, *OsbZIP23*, and *OsSIPP2C1* (ABA signal regulators) [27,28,29]. As such, we detected these five genes using qRT-PCR. As a result, except for *OsIAA15*, the expression of the other four genes showed a significant reduction in *m625* (Figure 6D). The data not only suggested the involvement of *NAL1* in regulating IAA and ABA signal transduction in the leaf but also validated the reliability of our RNA-Seq analysis. 

Furthermore, in the leaf at the heading stage, we identified 33 DEGs related to photosynthesis, which were primarily involved in photosynthesis, glyoxylate and dicarboxylate metabolism, carbon fixation in photosynthetic organisms, porphyrin and chlorophyll metabolism, and photosynthesis-antenna proteins (Figure 6B; Table S7). Among these DEGs, 27 genes were downregulated, including *OsCPL1* (chloroplastic-like protein) [30], *OsPsbS1* (photosystem II protein) [31], *OsPsbP* (polypeptide of the oxygen-evolving complex of photosystem II) [32], *OsPsbR3* (photosystem II polypeptide) [33], *OsFd1* (photosynthetic ferredoxin) [34], *OsRBCS3* and *OsRBCS4* (small subunits of Rubisco) [35]. The expression levels of several of them were examined by qRT-PCR and the results were consistent with the RNA-Seq analysis (Figure 6E), indicating the important role of *NAL1* in leaf photosynthesis.

**Figure 7:**
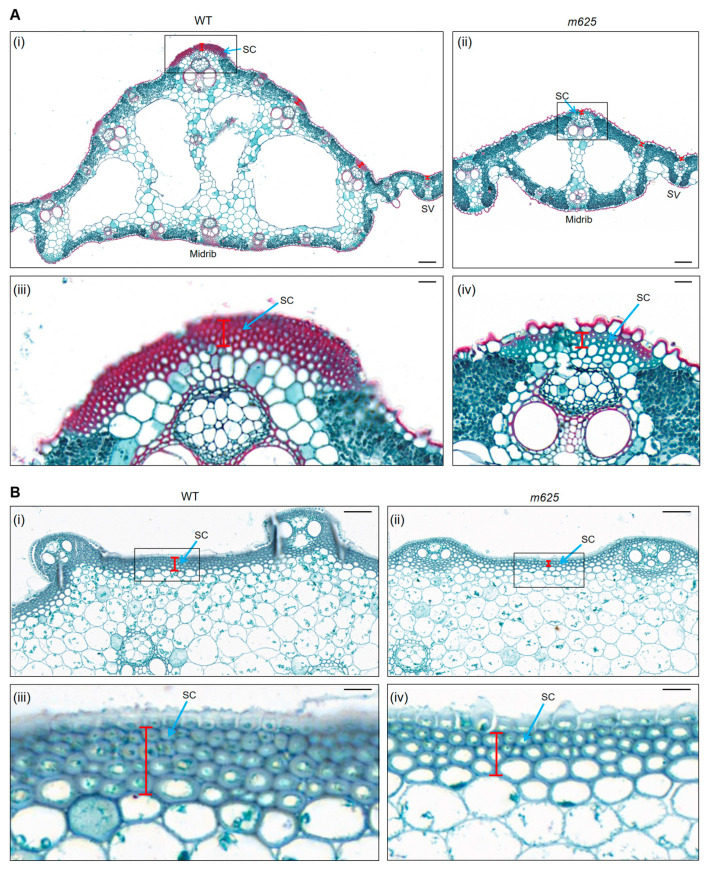
The analysis of sclerenchyma in the leaf and stem between WT and *m625*. (**A**) Comparison of sclerenchyma in the leaf at the heading stage. (**i**,**ii**) Sclerenchyma thickness in the leaf. (**iii**,**iv**) Enlarged images of sclerenchyma in the midrib. Scale bars, 100 μm (**i**,**ii**); 20 μm (**iii**,**iv**). (**B**) Comparison of sclerenchyma in the internode II at the heading stage. (**i**,**ii**) Sclerenchyma thickness in the stem. (**iii**,**iv**) Enlarged images of sclerenchyma in the internode II. Scale bars, 50 μm (**i**,**ii**); 10 μm (**iii**,**iv**). In sections, the lignified cell walls were stained red by saffron O staining solution in the midrib of the leaf. Blue arrows denote sclerenchyma cell. Red markers indicate sclerenchyma thickness. SC, sclerenchyma cell.

Additionally, we have made another finding that the DEGs in both leaf and stem at the heading stage were primarily associated with phenylpropanoid biosynthesis, specifically focusing on lignin biosynthesis (Figure 6B,C; Tables S7 and S8). Some of these genes are crucial for the formation and maintenance of cell walls. For example, *Os4CL2*, *Os4CL3* and *Os4CL5* encode 4-Coumarate:Coenzyme A Ligase, which participates in the phenylpropanoid metabolic pathway for the biosynthesis of monolignols and flavonoids. The enhanced expression of *Os4CL3* and *Os4CL5* significantly promotes the accumulation of lignin subunits G and S, leading to the strengthening of cell walls, particularly in the sclerenchyma thickness [36,37]. *OsCCR14* encodes a cinnamoyl–CoA reductase that affects lignification and the thickening of secondary cell walls in rice anthers and roots [38]. *OsPAL5*, *OsPAL6*, and *OsPAL7* are phenylalanine ammonia–lyase genes that regulate the biosynthesis and accumulation of lignin [39,40]. Therefore, we further examined the expressions of these genes, and found that all the detected genes were downregulated in the *m625* leaf and stem (Figure 6F,G). Considering that the accumulation of lignins directly affects the secondary cell wall formation and sclerenchyma thickness, we observed the sclerenchyma in the leaf and stem at the heading stage. Compared to the WT, the mutant *m625* displayed thinner sclerenchyma in both leaf and stem, with quantitative analysis confirming a significant decrease in sclerenchyma thickness (Figure 7 and Figure S4). Furthermore, a reduction in cell wall thickness was observed in the sclerenchyma cells of the *m625* leaf and stem, especially in the stem. In addition, a notable observation was less lignified cell walls in the midrib of the leaf in *m625* (Figure 7). Softening typically affects lignified staining under the same conditions, which may result in the absence of results for lignified cell wall staining in the stem. Although red stained lignified cells were not clearly observed in the stem, the existing findings have provided further support for the role of *NAL1* in lignification, secondary cell wall formation, and sclerenchyma thickness.

## 4. Discussion

*NAL1* (*LOC_Os04g52479*), which encodes a plant-specific protein, has been reported to regulate leaf size [10,41], leaf photosynthesis [42,43], leaf chlorophyll content [44], root development [11,45], plant height [10,45,46,47], large vascular bundle phloem area (LVPA) in the panicle neck [48], secondary branch number [49], and grain number and grain yield [45,49,50]. In previous studies, some allelic mutants and natural variants of *NAL1* have been identified in rice. Most of these natural variants exhibit normal stature in plants and have positive effects on many agronomic traits, including enhanced photosynthesis, increased flag leaf size, leaf width, panicle length, grain number, LVPA in the panicle neck, and regulation of source–sink–flow relationship [42,43,44,47,49]. Consequently, *NAL1* has been suggested as a valuable tool for improving photosynthesis and yield by manipulating leaf characteristics [31]. However, the precise mechanisms through which *NAL1* influences multiple biological processes in rice are still not fully understood. To gain a deeper understanding of how *NAL1* regulates rice growth and development, it is essential to identify additional allelic mutants of *NAL1* and explore novel regulatory pathways. 

Here, we discovered a novel allelic mutant of *NAL1*, *m625*, which exhibited pleiotropic phenotypes, including narrow leaf, dwarfism, multiple tillers, decreased panicle length, panicle branch number, and grain number per panicle (Figure 1 and Figure S1). Although the phenotypes of *m625* were similar to those previously reported in other *NAL1* allelic mutants, such as *nal1* (ZheFu802) [10], *nal1* (FL244) [11], *nal5* [11], *nal1-2* [46], and *nal1-3* [46], there were still some differences in mutation traits. By analyzing these subtle phenotypic variations in *m625*, we found that *NAL1* is not only involved in photosynthesis and IAA signaling, but also potentially plays important roles in ABA signaling, lignin biosynthesis, secondary cell wall formation, and sclerenchyma thickness.

### 4.1. NAL1 Regulate Leaf Photosynthesis

Several studies have emphasized the significance of *NAL1* in photosynthesis. Takanari (*indica* cv.) was shown to enhance mesophyll cell number, leaf thickness, leaf width, and photosynthesis rate by carrying the high-photosynthesis allele of *NAL1* [42]. Additionally, 9311-NIL with the Nipponbare *NAL1* exhibited increased leaf length, width, and chlorophyll content compared to 9311 [44]. A gradual decrease in leaf length and width was observed from 9311-*NIL* (full *NAL1* function) to 9311 (partial loss of *NAL1* function) and 9311-*nal1* (complete loss of *NAL1* function), while the leaf photosynthetic rate, expression of photosynthesis-related DEGs, and leaf thickness showed a gradual increase in the same order [43]. Meanwhile, we can clearly know from the above that the relationship between leaf dimensions (length, width, thickness) and photosynthesis is not consistently positive, as it is influenced by various factors.

Similarly, our results also provided evidence for the role of *NAL1* in controlling leaf photosynthesis. Specifically, the mutant *m625* exhibited reduced levels of photosynthetic pigments and downregulation of numerous photosynthesis-related DEGs (Figure 2 and Figure 6B; Table S7). However, our findings presented inconsistencies when compared to previously reported results that the partial or complete loss of *NAL1* function can increase expressions of photosynthesis-related DEGs [43]. Upon analysis, we hypothesized two potential reasons for this disparity. Firstly, previous studies usually analyzed RNA-Seq data from flag leaves, whereas our analysis focused on the top three leaves, which could be a contributing factor to the inconsistent outcomes. Secondly, it is important to note that *m625* is a *japonica* rice, whereas Takanari, 9311-NIL, 9311, and 9311-*nal1* are *indica* rice. In the *NAL1* gene, two rice subspecies have three SNP substitutions, leading to three amino acid changes, one of which affects the protein domain of *NAL1*, resulting in structural and functional differences [44]. In addition to *NAL1*, other upstream and downstream genes of *NAL1* in the photosynthesis pathway may also display genetic variations among different subspecies and cultivars. Furthermore, different mutation sites in *NAL1* mutants can alter protein structure, potentially influencing interactions with other proteins and overall protein functions. A comparison of Takanari, 9311-NIL, 9311, and 9311-*nal1* with *m625* revealed discrepancies in mutations within the *NAL1* gene. Takanari displayed 10 SNPs with 3 causing amino acid substitutions [42]. Fruthermore, 9311 has the same three amino acid substitutions, while 9311-NIL carried a Nipponbare *NAL1* from the japonica rice variety, and 9311-*nal1* showed a 1 bp insertion and a 1 bp deletion in the *NAL1* coding region, resulting in the loss of *NAL1* function [43,44]. On the other hand, the *m625* mutant presented a 1 bp deletion in the *NAL1* protein domain, causing premature termination of protein translation. Although *m625*, along with Takanari, 9311, and 9311-*nal1*, belongs to the *NAL1* function loss category, its genetic background and mutation site differ from them. Therefore, discrepancies in mutation sites and genetic backgrounds in *m625* may be the primary reasons for the inconsistent results.

### 4.2. NAL1 May Be Involved in IAA and ABA Signal Transduction to Regulate Rice Growth and Development

Plant hormones are known to regulate various physiological and developmental processes in rice. Mutations of *NAL1* have been identified to affect polar auxin transport activity [10,11]. However, in this study, our results suggested a novel finding that *NAL1* may be involved in regulating the ABA signal transduction in addition to IAA. RNA-Seq and qRT-PCR analyses revealed that the transcript levels of AUX/IAA genes (*OsIAA2*, *OsIAA15*) and ABA signal regulators (*OsPP2C09*, *OsbZIP23*, and *OsSIPP2C1*) were reduced in *m625* (Figure 6A,D; Table S6). As early IAA response genes in IAA signaling, AUX/IAA regulate the expression of auxin-response genes by interactions with ARFs [51,52], and the IAA and ABA signaling genes also experience significant crosstalk. Furthermore, *OsPP2C09* exhibits PP2C phosphatase activity, and its mutation inhibits growth, including plant height and panicle length [27]. *OsbZIP23* is a transcriptional regulator, and its overexpression shows a significant improvement in yield-related traits [28,53]. *OsSIPP2C1* is negatively regulated by *ABL1* and is responsible for panicle development in rice [29]. Based on the above analyses, we propose that the reduced expression levels of these genes may contribute to the phenotypes of narrow leaves, dwarfism, and small panicles in *m625*. Additionally, NAL1 protein has been identified to degrade FZP and OsTPR2. Reducing FZP expression or enhancing NAL1 expression have been shown to increase secondary branch number, grain number, and grain yield [49]. Mutations in NAL1 protein result in elevated OsTPR2 expression and reduced expression of downstream genes associated with IAA and strigolactone (SL) signaling, leading to narrower flag leaf, decreased plant height, lower grain number, and reduced grain yield [45]. Therefore, as a pleiotropic gene, *NAL1* is likely to be involved in IAA and ABA signal transduction to regulate rice growth and development.

### 4.3. NAL1 May Regulate the Secondary Cell Wall Formation and Sclerenchyma Thickness through Lignification 

Compared to previous studies, our research has made another novel discovery regarding the function of *NAL1*. In paraffin sections, we observed that the mutant *m625* exhibited thinner sclerenchyma and cell walls in the leaf and stem, with particularly less lignified cell walls in the midrib of the leaf (Figure 7). Additionally, RNA-Seq analysis showed that a majority of DEGs related to lignin biosynthesis were downregulated, which was further confirmed through qRT-PCR (Figure 6B,C,F,G; Tables S7 and S8). These findings strongly indicate that *NAL1* likely plays a crucial role in regulating secondary cell wall formation and sclerenchyma thickness by influencing lignin biosynthesis. Previous studies have demonstrated that several genes controlling leaf shape can regulate cell wall formation, such as *NRL1* [15,16], *NRL2* [17], and *SRL1* [54]. However, apart from previous RNA-Seq analysis briefly mentioning that *NAL1* may regulate cell wall formation [41,43], there are no detailed reports that *NAL1* regulates the secondary cell wall formation and sclerenchyma thickness through lignification. Therefore, our findings provide clear evidence for further studies on the regulatory role of *NAL1* in this pathway.

## 5. Conclusions

*m625* is a novel allelic mutant of *NAL1* which exhibited pleiotropic traits, including narrower leaves, dwarfism, increased tillers, and decreased panicle length, panicle branch number, and grain number. A nucleotide deletion in the fourth exon of *NAL1* in *m625* affected photosynthetic pigment levels, vascular patterns, and cell division. Through RNA-Seq analysis, this study revealed two new insights into the function of *NAL1*. Firstly, besides its previously known role in IAA transport, *NAL1* may also be involved in ABA signal transduction to regulate rice growth and development. Secondly, *NAL1* may play a role in regulating secondary cell wall formation and sclerenchyma thickness through lignification, as supported by cytological analysis. These findings contribute to a better understanding of the multifaceted functions of *NAL1*.

## Data Availability

The data presented in this study are available on request from the corresponding author Yang Wang. The data are not publicly available due to privacy restrictions.

## Supplementary Materials

The following supporting information can be downloaded at: https://www.mdpi.com/article/10.3390/genes15030325/s1, Figure S1: Comparison of major agronomic traits of wild-type (WT) and *m625* mutant; Figure S2: Comparison of leaf length, leaf width, and plant height of WT and TP; Figure S3: Venn analysis of DEGs; Figure S4: Comparison of sclerenchyma thickness in the leaf and stem between WT and *m625*; Table S1: Primers used in gene mapping, amplification sequencing, vector construction and detection; Table S2: Primers used in qRT-PCR; Table S3: Segregation of F_2_ populations from the crosses between *m625* and wild type (WT); Table S4: Putative genes within the 167.8 kb region; Table S5: The number of DEGs in *m625* compared to the WT; Table S6: DEGs associated with plant hormone signal transduction and plant-pathogen interaction pathways in the leaf at the tillering stage; Table S7: DEGs associated with photosynthesis and phenylpropanoid biosynthesis pathways in the leaf at the heading stage; Table S8: DEGs associated with phenylpropanoid biosynthesis and phenylalanine metabolism pathways in the stem at the heading stage.
