# Supplementary material for: Identification and Transcriptome Analysis of a Novel Allelic Mutant of NAL1 in Rice
Source: Genes (Basel). 2024 Mar 2;15(3):325. doi: 10.3390/genes15030325 (PMC10970654; doi:10.3390/genes15030325)
Supplement: Supplementary file 1 [file genes-15-00325-s001.zip › Supporting Information FigS1-S4.pdf]

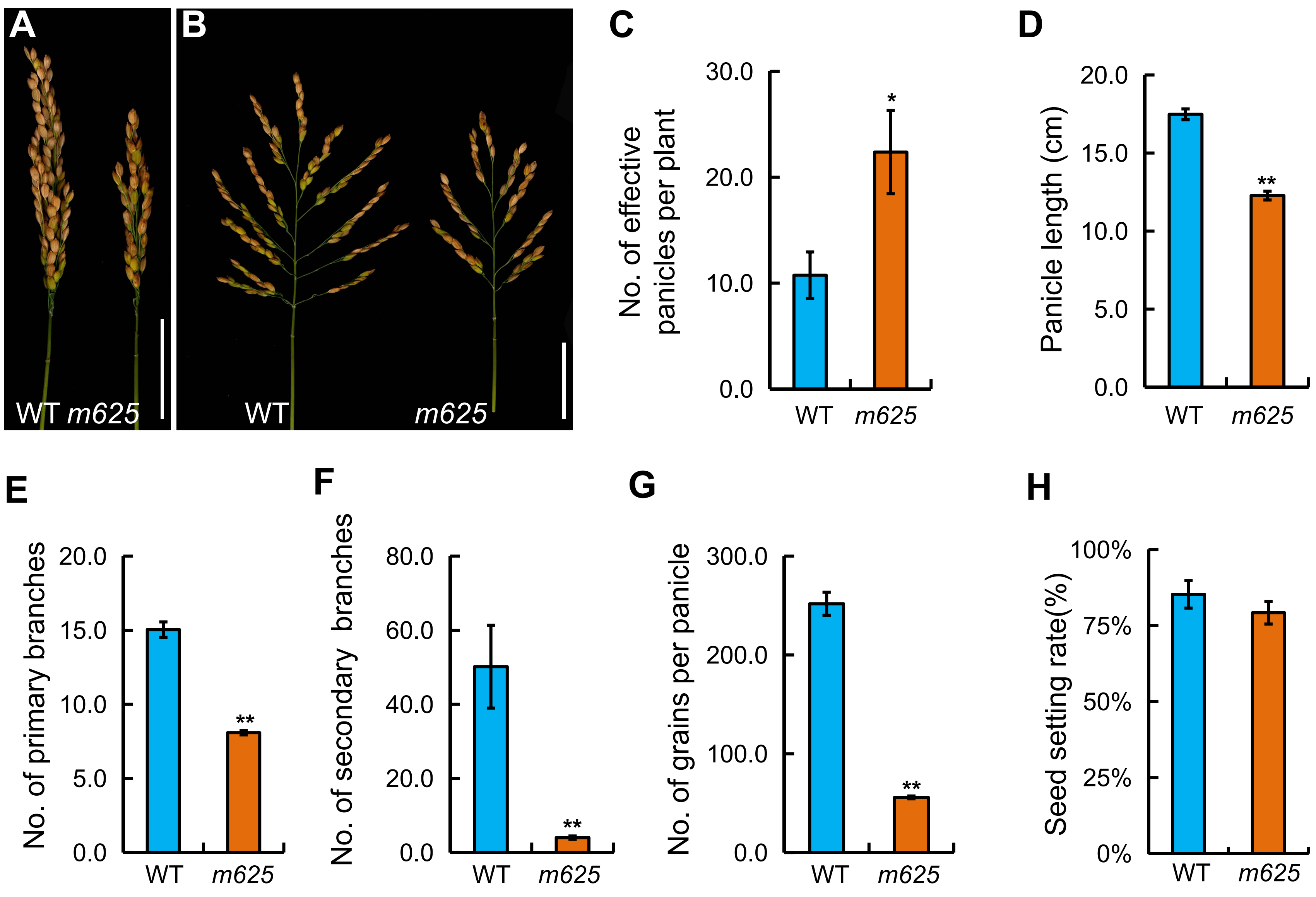

Figure S1. Comparison of major agronomic traits of wild type (WT) and *m625* mutant. (**A**,**B**) Panicle architecture of WT and *m625*. Scale bars, 5 cm. (**C**-**H**) Statistical analysis of effective panicle number per plant, panicle length, primary branch number, secondary branch number, grain number per panicle and setting rate. The blue and orange columns represent WT and *m625* in the histogram, respectively, the same as below. Data are means ± SD (n=24). Asterisks indicate statistically significant differences by a Student's t-test ($^{*}P < 0.05$; $^{**}P < 0.01$).

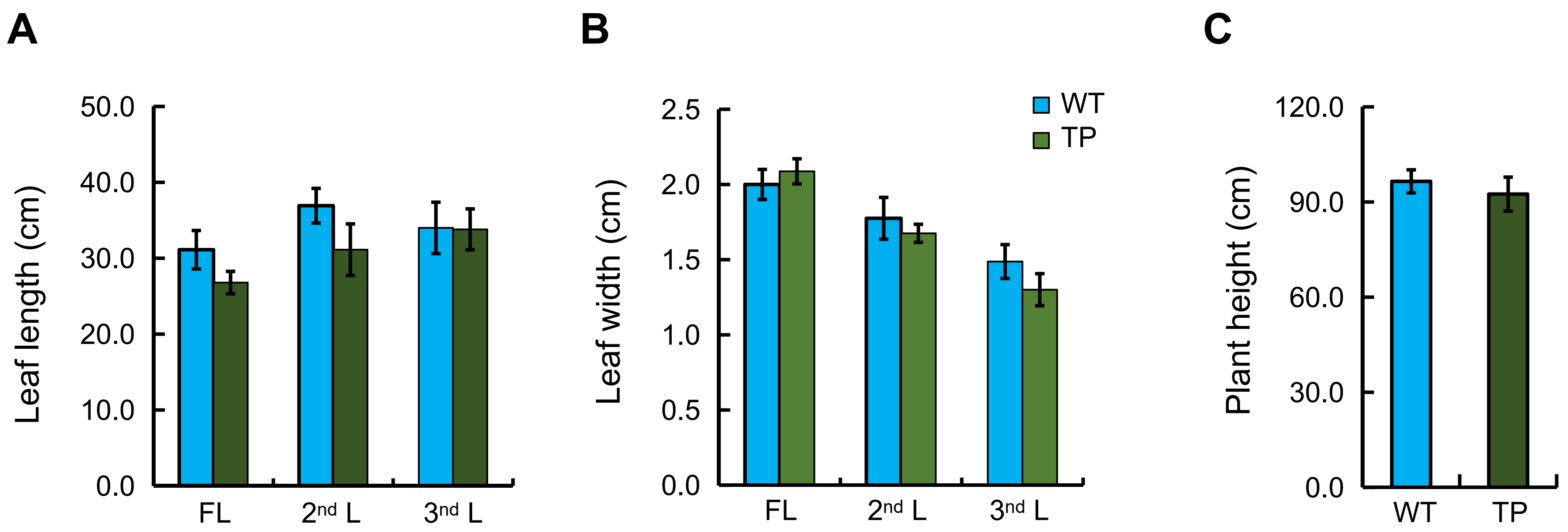

Figure S2. Comparison of leaf length, leaf width and plant height of WT and TP. (**A**) Leaf length. (**B**) Leaf width. (**C**) Plant height. The blue and green columns represent WT and *m625* in the histogram, respectively. Data are means ± SD (*n*=6).

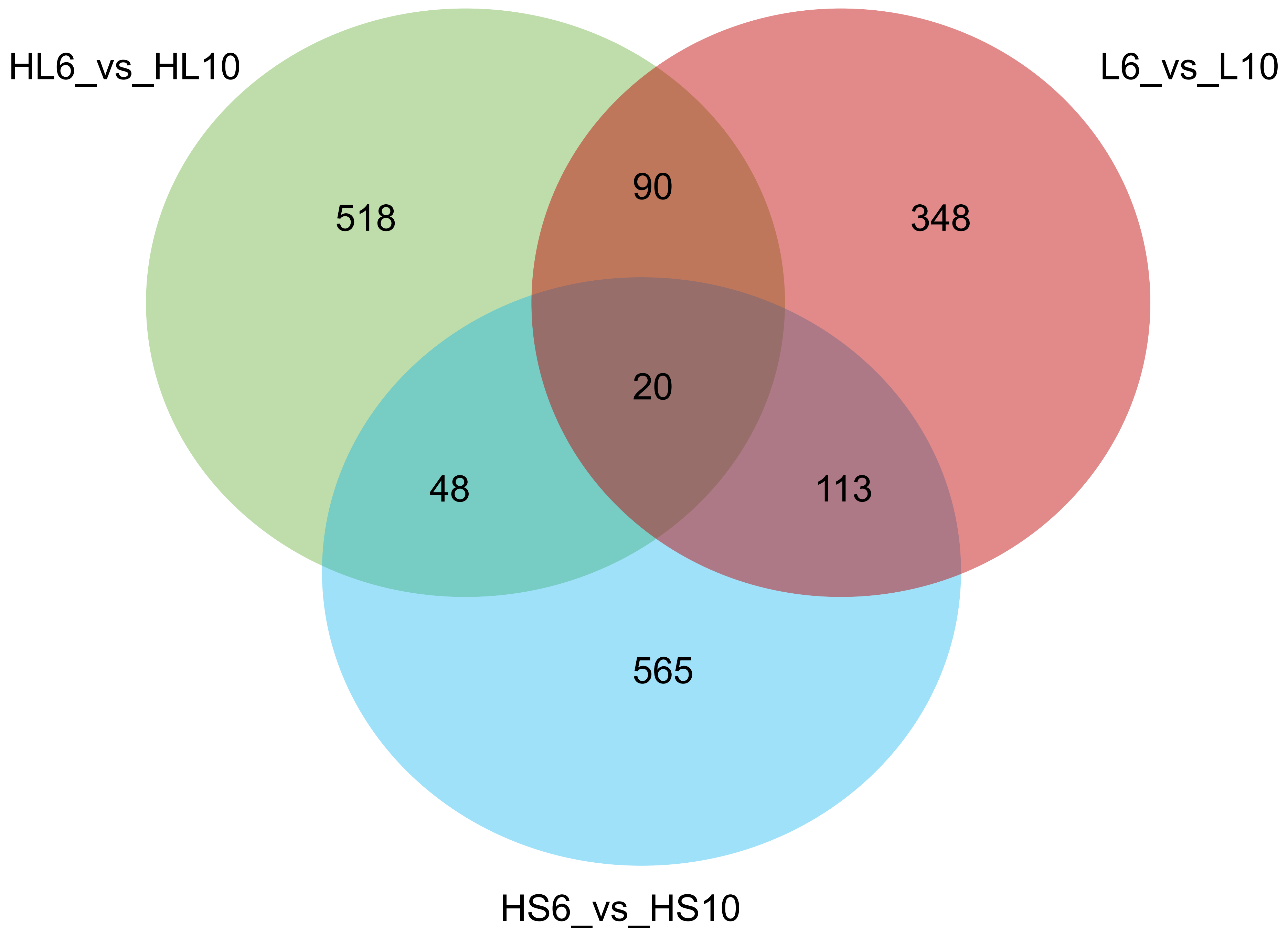

Figure S3. Venn analysis of DEGs. L6 and L10 are the WT leaf and *m625* leaf at the tillering stage, HL6 and HL10 are the WT leaf and *m625* leaf at the heading stage, HS6 and HS10 are the WT stem and *m625* stem at the heading stage, respectively.

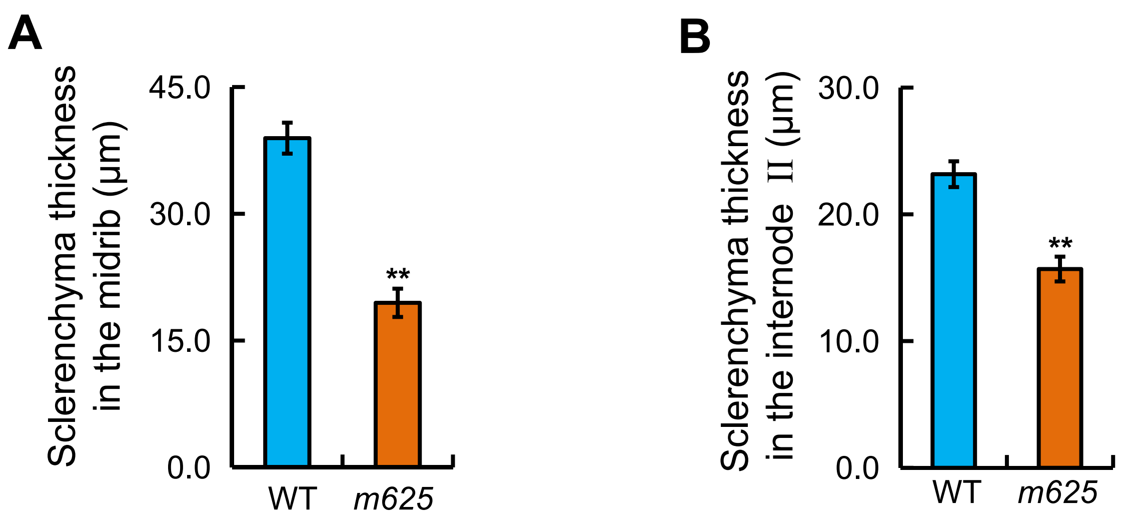

Figure S4. Comparison of sclerenchyma thickness in the leaf and stem between WT and *m625*. (**A**) Comparison of sclerenchyma thickness in the midrib of the leaf at the heading stage. (**B**) Comparison of sclerenchyma thickness in the internode II at the heading stage. Data are means ± SD from three biological replicates. Asterisks represent significant differences using Student's *t*-test (** $p < 0.01$).
