# Supplementary material for: Identification and Transcriptome Analysis of a Novel Allelic Mutant of NAL1 in Rice
Source: Genes (Basel). 2024 Mar 2;15(3):325. doi: 10.3390/genes15030325 (PMC10970654; doi:10.3390/genes15030325)
Supplement: Supplementary file 1 [file genes-15-00325-s001.zip › Supporting Information Table S1-S8.pdf]

Table S1. Primers used in gene mapping, amplification sequencing, vector construction and detection.

Purpose	Primer name	Forward primer (5'-3')	Reverse primer (5'-3')
Gene mapping	RM241	GAGCCAAATAAGATCGCTGA	TGCAAGCAGCAGATTTAGTG
	RM303	GCATGGCCAAATATTAAAGG	GGTTGGAAATAGAAGTTCGGT
	RM349	TTGCCATTCGCGTGGAGGCG	GTCCATCATCCCTATGGTCG
	N1	ATGACTGATCCTTGTCTTC	GATCGTGATTCCATGATG
	N2	AAGAGCTACTAGGTACCT	CTCATTATTAGTGGTGGC
	N3	CAGGAGAGATATTACACA	CCGATGATATCTGCAAGC
DNA sequencing	*M625*-1	ATCTACCTGTCCATTAGCC	GTGCCCTTCCTCTTGCCA
	M625-2	AGCGGAGTTGGCAGCAGCCA	TCGAACTCGGTGAAGAACT
	M625-3	AGCTCACGCAAGCATCGA	CACGCTCGGTGAAGAAGAGCT
	M625-4	AGACACAGCGTGTGCGCATTA	TCGTCGGTCGCTGCATGAA
	M625-5	ACCTTCCAGATGAGTGATCT	AAGAGGATGGCACTCGAGAT
	M625-6	TTGGATGCTCTAGGACAG	CTATAAGGACGAACGCTTCA
	M625-7	ACAATCCTCCTACCGTTTACC	CTTGGAGTGATTCATTGGT
	M625-8	ACACAACTGGGACTGTGATGG	CTGCAGGACTAGCACCTAA
cDNA sequencing	*M625*-C1	ATGAAGCCTTCGGACGATAA	GGACACTGCAGATCTATAA
	M625-C2	ACTACCCTAATCAGAAGATG	TCATTTCTCCAGGTCAAGGC
Complementation	*M625*-Co	GGTACCCGGGGATCCCTGCAGATGAAGCCTTCGGACGATAA	TGCCTGCAGGTCGACCTGCAGTCATTTCTCCAGGTCAAGGC
Detection	*M625*-Tra	CTACTCGACCGTCTGGAAC	GCGATCATAGGCGTCTCG

Table S2. Primers used in qRT-PCR.

Marker	Forward primer (5′-3′)	Reverse primer (5′-3′)
OsIAA2	GATGTTCATCGCCACCGC	CTGCTGCCCTCTTCCTGC
OsIAA15	TCGGGAACGAGGAGATGA	TGGCAGGTCTCCACAAACA
OsPP2C09	GCCTTGGAGGGATGTGATGG	CGAAGGCGTCTGCTGCTC
OsbZIP23	AACGATGAACTCCAGAAA	AGTCCTCCGAAGGCAAAT
OsSIPP2C1	GGGAGGAGGAGGGAGATGGA	CGAACACCCCGTAGAAGTCACA
OsCPL1	GTGGTGTTCGACGAGGACG	TGAGCGTGACGGAGAAGGT
OsPsbS1	GTCGCCATGCTCGGGTTT	GGGCTCCGCCTCGTAGAT
OsPsbP	ATAATCAACCCCACCACC	ATCTGTCTTGCCGGAGTAG
OsFd1	GCACTGAGCAGCCAGGTCC	ATCCGCAGCCTGTCTCCC
Os4CL2	CAGCAGGTGGACGGCGAGAA	CAGCACGGAGTTGAGCGAGAAG
OsPAL5	ATGTTCGCCCAGTTCTCCG	GGTGCCCTTGAAGCCGTAG
OsPAL6	GGCGAGGAGGTGAACAAGG	CAGCAGATGGGCAGAGGC
Os4CL3	CCTGAGGCGACCAAGAAC	GCCTGTCCACGATGAAGAT
Os4CL5	ATCCCAGTGGCGTTCGTC	TCTTGTGCAGCCTCTTGTAG
OsCCR14	CCTCTGCGCCGAGTCTGT	GCTGCTTCCTGGGGTTCTT
OsPAL7	CCGAGCAGCACAACCAGGAC	CGACGAGGAAGGTGGAGGACA
Actin 1	TGTATGCCAGTGGTCGTACCA	CCAGCAAGGTCGAGACGAA

Table S3. Segregation of F_2 populations from the crosses between *m625* and wild type (WT).

Combination	No. of total plants	No. of normal leaf plants	No. of narrow leaf plants	Expected ratio	χ^2	$\chi^2_{0.05}$
m625 / Qiaogangzhenzhu	484	368	116	3:1	0.27	3.84

Table S4. Putative genes within the 167.8-kb region.

Number	MSU Locus ID	Gene function annotation
ORF1	*LOC_Os04g52440*	Aminotransferase, putative, expressed
ORF2	*LOC_Os04g52450*	γ-aminobutyric acid (GABA):pyruvate transaminase
ORF3	*LOC_Os04g52460*	Retrotransposon protein, putative, unclassified, expressed
ORF4	*LOC_Os04g52479*	Trypsin-like serine/cysteine protease
ORF5	*LOC_Os04g52500*	Lecithine cholesterol acyltransferase, putative, expressed
ORF6	*LOC_Os04g52504*	Adhesive/proline-rich protein, putative, expressed
ORF7	*LOC_Os04g52510*	Glucosyltransferase, putative, expressed
ORF8	*LOC_Os04g52520*	APO, putative, expressed
ORF9	*LOC_Os04g52530*	Heavy metal-associated domain containing protein, expressed
ORF10	*LOC_Os04g52540*	ARGONAUTE family protein
ORF11	*LOC_Os04g52550*	PAZ domain-containing protein, putative, expressed
ORF12	*LOC_Os04g52560*	transposon protein, putative, unclassified, expressed
ORF13	*LOC_Os04g52570*	expressed protein
ORF14	*LOC_Os04g52580*	Tetratricopeptide repeat domain containing protein, expressed
ORF15	*LOC_Os04g52590*	Protein kinase domain containing protein, expressed
ORF16	*LOC_Os04g52600*	SHR5-receptor-like kinase, putative, expressed
ORF17	*LOC_Os04g52606*	SHR5-receptor-like kinase, putative, expressed
ORF18	*LOC_Os04g52614*	SHR5-receptor-like kinase, putative, expressed
ORF19	*LOC_Os04g52630*	Leucine-rich repeat-containing protein kinase family protein, putative, expressed
ORF20	*LOC_Os04g52640*	SHR5-receptor-like kinase, putative, expressed
ORF21	*LOC_Os04g52650*	expressed protein
ORF22	*LOC_Os04g52660*	expressed protein

Table S5. The number of DEGs in *m625* compared to the WT.

Control (WT)	Treat (*m625*)	Tissue	Stage	Up-regulated DEGs	Down-regulated DEGs	Total
L6	L10	Leaf	Tillering stage	164	407	571
HL6	HL10	Leaf	Heading stage	310	366	676
HS6	HS10	Stem	Heading stage	469	277	746

Table S6. DEGs associated with plant hormone signal transduction and plant-pathogen interaction pathways in the leaf at the tillering stage.

Pathway	No. of DEGs	Gene ID	log2FoldChange (L10/L6)	Up-regulated or Down-regulated	Gene function annotation
Plant hormone signal transduction	8	*LOC_Os01g55940*	4.3	Up	*OsGH3-2*; indole-3-acetic acid-amido synthetase gene
		LOC_Os05g08570	-2.2	Down	OsIAA15-Auxin-responsive Aux/IAA gene family member, expressed
		LOC_Os01g28500	-3.0	Down	SCP-like extracellular protein, expressed
		LOC_Os01g09450	-1.4	Down	OsIAA2-Auxin-responsive Aux/IAA gene family member, expressed
		LOC_Os01g62760	-1.4	Down	*OsPP2C09;* type 2C protein phosphatase; PP2C protein
		LOC_Os01g40094	-1.7	Down	*OsABIL1;* clade A type 2C protein phosphatase
		LOC_Os02g52780	-1.6	Down	*OsbZIP23;* bZIP transcription factor
		LOC_Os09g15670	-3.9	Down	*OsSIPP2C1*; Protein Phosphatase 2C
Plant-pathogen interaction	8	*LOC_Os05g42250*	1.0	Up	*OsCNGC16*, cyclic nucleotide-gated ion channel protein
		LOC_Os04g41540	-1.2	Down	*OsCML22*; OsCML22-Calmodulin-related calcium sensor protein, expressed
		LOC_Os05g31620	-5.6	Down	*OsCML15*; OsCML15-Calmodulin-related calcium sensor protein, expressed
		LOC_Os03g19720	-1.1	Down	EF hand family protein, putative, expressed
		LOC_Os01g04330	-3.7	Down	*OsCML16*; OsCML16-Calmodulin-related calcium sensor protein, expressed
		LOC_Os01g72080	-1.3	Down	Calmodulin-like protein 1, putative, expressed
		LOC_Os01g28500	-3.0	Down	SCP-like extracellular protein, expressed
		LOC_Os01g72530	-8.7	Down	*OsMSR2*; calmodulin-like gene

Table S7. DEGs associated with photosynthesis and phenylpropanoid biosynthesis pathways in the leaf at the heading stage.

Pathway	No. of DEGs	Gene ID	log2FoldChange (HL10/HL6)	Up-regulated or Down-regulated	Gene function annotation
Photosynthesis	15	*LOC_Os07g05360*	3.9	Up	*OsPsbR1*; 10 kDa Photosystem II polypeptide
		LOC_Os07g01480	-2.2	Down	Oxygen evolving enhancer protein 3 domain containing protein, expressed
		LOC_Os07g05480	-2.1	Down	Photosystem I reaction center subunit, chloroplast precursor
		LOC_Os06g01210	-2.1	Down	*OsCPL1*; chloroplastic-like protein
		LOC_Os03g21560	-3.6	Down	Photosystem II 11 kD protein, putative, expressed
		LOC_Os04g33830	-2.0	Down	Membrane protein, putative, expressed
		LOC_Os02g51470	-2.2	Down	ATP synthase F1, delta subunit family protein, putative, expressed
		LOC_Os01g64960	-3.2	Down	*OsPsbS1*; photosystem b
		LOC_Os07g04840	-1.8	Down	*OsPsbP*; Polypeptide of the Oxygen-Evolving Complex of Photosystem II
		LOC_Os07g36080	-1.8	Down	Oxygen evolving enhancer protein 3 domain containing protein, expressed
		LOC_Os08g10020	-1.8	Down	*OsPsbR3*; 10 kDa Photosystem II polypeptide
		LOC_Os01g71190	-2.5	Down	Photosystem II reaction center PSB28 protein, chloroplast precursor
		LOC_Os05g48630	-2.0	Down	Expressed protein
		LOC_Os08g01380	-2.1	Down	*OsFd1*; photosynthetic ferredoxin
		LOC_Os12g23200	-1.9	Down	Photosystem I reaction center subunit XI, chloroplast precursor
Phenylpropanoid biosynthesis	10	*LOC_Os03g13210*	4.4	Up	*OsPRX38*; class III peroxidase
		LOC_Os01g73170	3.3	Up	*Perox3*; peroxidase
		LOC_Os07g46280	3.2	Up	*Os7BGlu26*; *β*-D-glucosidase; GH1 glycoside hydrolase
		LOC_Os02g46970	-4.5	Down	*Os4CL2*; 4-Coumarate:Coenzyme A Ligase
		LOC_Os04g55740	-Inf	Down	Peroxidase precursor, putative, expressed
		LOC_Os04g43760	-1.9	Down	*OsPAL5*; phenylalanine ammonia-lyase gene
		LOC_Os10g42800	-2.1	Down	AMP-binding enzyme, putative, expressed
		LOC_Os01g40860	-3.6	Down	*OsALDH2C4*; *ALDH1a; OsALDH2-1*; aldehyde dehydrogenase

		LOC_Os04g43800	-4.7	Down	*OsPAL6*; phenylalanine ammonia-lyase gene
		LOC_Os03g49600	-4.1	Down	*Os3BGlu7*; *bglu1*; *β*-glucosidase
Glyoxylate and dicarboxylate metabolism	9	*LOC_Os07g34520*	1.1	Up	Isocitrate lyase, putative, expressed
		LOC_Os04g40990	9.2	Up	Malate synthase, glyoxysomal, putative, expressed
		LOC_Os01g02020	2.0	Up	Acetyl-CoA acetyltransferase, cytosolic, putative, expressed
		LOC_Os10g37180	-4.0	Down	*OsGDCH*; glycine decarboxylase complex H-protein
		LOC_Os06g40940	-2.4	Down	Lycine dehydrogenase, putative, expressed
		LOC_Os04g53230	-3.3	Down	Aminomethyltransferase, putative, expressed
		LOC_Os01g22520	-1.9	Down	Dihydrolipoyl dehydrogenase 1, mitochondrial precursor
		LOC_Os12g19381	-2.5	Down	*OsRBCS3*; small subunit of Rubisco
		LOC_Os12g19470	-3.2	Down	OsRBCS4; small subunit of Rubisco
Carbon fixation in photosynthetic organisms	5	*LOC_Os05g41640*	-2.8	Down	Phosphoglycerate kinase protein, putative, expressed
		LOC_Os12g19381	-3.2	Down	*OsRBCS3*; small subunit of Rubisco
		LOC_Os12g19470	-2.5	Down	*OsRBCS4*; small subunit of Rubisco
		LOC_Os07g08030	-1.8	Down	Ribose-5-phosphate isomerase A, putative, expressed
		LOC_Os06g40640	-2.0	Down	*OsAld-Y*; *ygdl-1*; fructose-1,6-bisphosphate aldolase
Porphyrin and chlorophyll metabolism	4	*LOC_Os10g25030*	3.4	Up	Red chlorophyll catabolite reductase, putative, expressed
		LOC_Os10g25040	5.3	Up	Red chlorophyll catabolite reductase, putative, expressed
		LOC_Os04g59610	-1.9	Down	*OsSGRL*; Stay-Green Rice like gene
		LOC_Os03g36540	-1.9	Down	*OsCHLI*; magnesium-chelatase subunit ChlI
Photosynthesis-antenna proteins	2	*LOC_Os01g52240*	-3.3	Down	Chlorophyll A-B binding protein, putative, expressed
		LOC_Os07g37240	-3.9	Down	Chlorophyll A-B binding protein, putative, expressed

Table S8. DEGs associated with phenylpropanoid biosynthesis and phenylalanine metabolism pathways in the stem at the heading stage.

Pathway	No. of DEGs	Gene ID	log2FoldChange (HS10/HS6)	Up-regulated or Down-regulated	Gene function annotation
Phenylpropanoid biosynthesis	12	*LOC_Os07g46280*	2.2	Up	*Os7BGlu26*; β-D-glucosidase; GH1 glycoside hydrolase
		LOC_Os02g08100	-1.0	Down	*Os4CL3*; 4-Coumarate:Coenzyme A Ligase
		LOC_Os02g26810	-1.9	Down	Cytochrome P450, putative, expressed
		LOC_Os08g34280	-1.0	Down	*OsCCR14*; *OsCCR20*; cinnamoyl-CoA reductase
		LOC_Os05g25640	-1.2	Down	Cytochrome P450, putative, expressed
		LOC_Os04g43760	-1.2	Down	*OsPAL5*; phenylalanine ammonia-lyase gene
		LOC_Os05g04500	-1.1	Down	Peroxidase precursor, putative, expressed
		LOC_Os01g73170	-1.2	Down	*Perox3*; peroxidase
		LOC_Os01g18110	-1.4	Down	Cinnamoyl CoA reductase, putative, expressed
		LOC_Os08g34790	-1.6	Down	*Os4CL5*; 4-Coumarate:Coenzyme A Ligase
		LOC_Os11g02130	-1.2	Down	Peroxidase precursor, putative, expressed
		LOC_Os05g35290	-1.2	Down	*OsPAL7*; phenylalanine ammonia-lyase gene
Phenylalanine metabolism	7	*LOC_Os05g25640*	-1.2	Down	Cytochrome P450, putative, expressed
		LOC_Os04g43760	-1.2	Down	*OsPAL5*; phenylalanine ammonia-lyase gene
		LOC_Os08g04560	-4.2	Down	*OsTDC3*; tryptophan decarboxylase
		LOC_Os05g35290	-1.2	Down	*OsPAL7*; phenylalanine ammonia-lyase gene
		LOC_Os02g08100	-1.0	Down	*Os4CL3*; 4-Coumarate:Coenzyme A Ligase
		LOC_Os08g34790	-1.6	Down	*Os4CL5*; 4-Coumarate:Coenzyme A Ligase
		LOC_Os02g26810	-1.9	Down	Cytochrome P450, putative, expressed
